# Livestock infected with *Leishmania* spp. in southern Iran

**DOI:** 10.1186/s13071-022-05313-8

**Published:** 2022-06-17

**Authors:** Zahra Rezaei, Bahman Pourabbas, Sadaf Asaei, Shima Sepehrpour, Sara Ahmadnia Motlagh, Parham Pourabbas, Samaneh Abdolahi Khasibi, Abdolvahab Alborzi

**Affiliations:** 1 Professor Alborzi Clinical Microbiology Research Center, Shiraz University of Medical Sciences, Shiraz, Iran; 2 Department of Parasitology and Mycology, Faculty of Medicine, Zahedan University of Medical Sciences, Zahedan, Iran

**Keywords:** Iran, Leishmaniasis, *Leishmania* infection, Livestock, Reservoirs

## Abstract

**Background:**

Query ID="Q1" Text="Graphical abstract: As per journal requirements, graphical abstract is necessary. Kindly check and provide the same."The magnitude of the health problems caused by leishmaniasis has been a major driving factor behind the development and implementation of leishmaniasis control programs by the national authorities in Iran, with a priority for health and environmental management. Such programs are not achievable unless all of the factors leading to the infection, including the parasite’s life-cycle, vectors and reservoirs, are recognized. So far in Iran, humans and rodents have been considered the principal reservoirs of *Leishmania tropica* and *Leishmania major*, respectively, both associated with cutaneous leishmaniasis (CL), with domestic dogs considered to be the main reservoir for *Leishmania infantum,* associated with visceral leishmaniasis (VL). The role of other mammals in maintaining the *Leishmania* parasite has remained unclear. This study aimed to investigate *Leishmania* infection among livestock in endemic areas of VL and CL in Fars province, southern Iran, using serological and molecular methods.

**Methods:**

Blood samples from 181 clinically healthy livestock, including 49 sheep, 114 goats, 16 cattle and two donkeys, were screened to detect *Leishmania* DNA and anti-*Leishmania* antibodies using qPCR (quantitative PCR) and the direct agglutination test (DAT), respectively. Four qPCR-positive samples were amplified using the internal transcribed spacer one (ITS1) primers in conventional PCR and sent for directional sequencing.

**Results:**

Of the 181 livestock tested, 51 (28.2%) were infected with *Leishmania*, using serological and molecular methods. Anti-*Leishmania* antibodies were detected in 70 (38.7%) (95% confidence interval [CI]: 31.5–46.2) and *Leishmania* DNA in 93 (51.4%) (95% CI: 43.9–58.9) livestock. The identified *Leishmania* spp. were *L. infantum* and *L. major*.

**Conclusions:**

The findings of the present study show a relatively high prevalence of *Leishmania* infection among livestock in endemic areas of the disease, in Fars province, southern Iran. Given the large population of this group of animals and the fact that they live in the vicinity of the main reservoirs of the disease and vectors, it seems that sand flies regularly bite these animals. Further studies are needed to determine the role of livestock in the parasite’s life-cycle and the epidemiology of *Leishmania* infection.

**Graphical Abstract:**

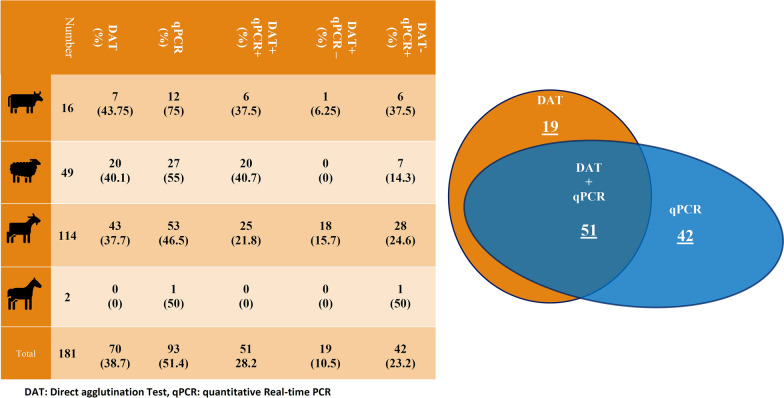

## Background

Leishmaniasis is a vector-borne disease caused by obligate intracellular parasites in the genus *Leishmania* that can be classified by geographic occurrence into Old World and New World *Leishmania* species. Old World leishmaniasis can lead to cutaneous leishmaniasis (CL), caused by *Leishmania major*, *L. tropica* and *L. aethiopica*, and visceral leishmaniasis (VL), caused by *Leishmania infantum* and *L. donovani* [1]. The WHO estimated that 350 million persons in 98 countries, including Iran, are at the risk of developing leishmaniasis, with an annual incidence of around 1.5 million new cases [2].

Iran is one of the countries included in the WHO Eastern Mediterranean Region, and accounts for 54% of CL cases in that region [3]. Although the incidence of VL cases is lower than that of CL, VL remains a major health concern in the respective endemic areas. In Iran, CL is caused by *L. major* and *L. tropica*, whereas VL is caused by *L. infantum* and, to a lesser extent, *L. tropica*.

*Leishmania* infection can be anthroponotic, i.e., transmitted from human to human, or zoonotic, with wild or domestic animals as the reservoir [4]. Mammals that can sustain the population of the infectious agents are called reservoirs and play an essential role in maintaining the life-cycle of the organism and, thus, the continued survival of the infectious agents [5, 6]. In addition to the main reservoirs, some incidental hosts can temporarily maintain the parasite and play a role in transmitting the infection [5]. Determination of the life-cycle of parasites and identification of sand fly vectors and reservoirs play a crucial role in obtaining a better understanding of epidemiology and ultimately applying appropriate strategies to control and eliminate the disease. The magnitude of leishmaniasis-associated problems, a disease which mainly affects poor communities due to the lack of preventive measures, has made the characterization of the above-mentioned issues more necessary. The principal reservoirs of *L. tropica* and *L. major* are humans and rodents, respectively [7]; however, the zoonotic transmission of *L. tropica* has been reported in some regions [8, 9]. Domestic dogs are considered to be the main reservoir for *L. infantum,* but other mammals have also been shown to be able to sustain the parasite [10, 11]. Previous epidemiological studies have *Leishmania* infection in horses, goats, cattle and cows in endemic regions of the disease, and some studies have shown that *Leishmania* vectors (Phlebotomine) bite these groups of animals and feed on their blood [12–17]. However, the roles of such mammals in maintaining infection and functioning as reservoirs have not yet been investigated.

To our knowledge, there has been no study in Iran investigating livestock infection caused by *Leishmania* spp. Therefore, the aim of the present study was to evaluate *Leishmania* infection in livestock in VL and CL endemic regions of southern Iran.

## Methods

### Study site and blood sampling

**Fig. 1:**
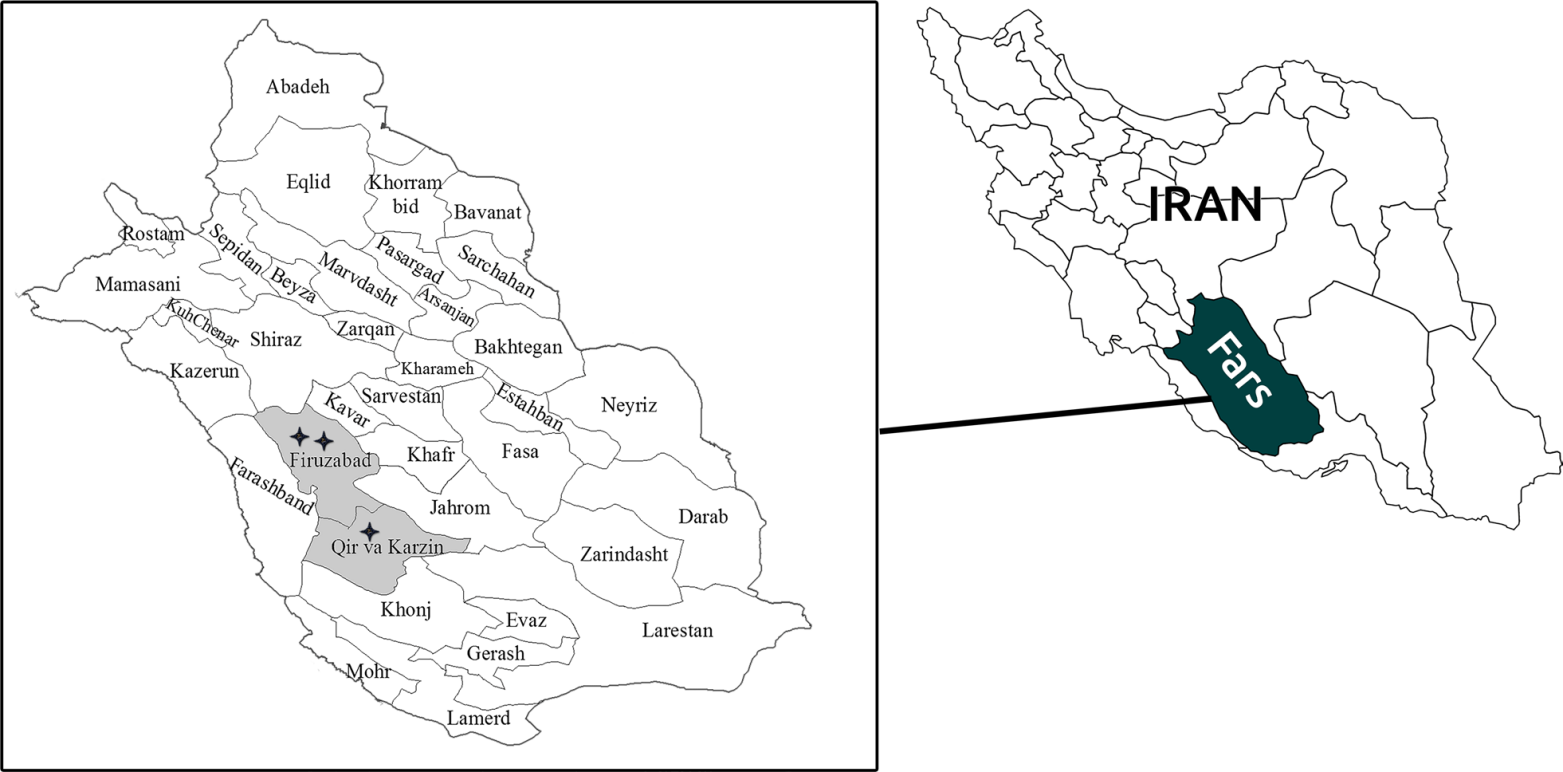
The geographical location of the study on a map of Fars province

This seroepidemiological and molecular study of infection with *Leishmania* in livestock was carried out in three villages in Fars province, southern Iran, in VL and CL endemic regions. The study areas are located in a semi-arid region. Two villages, Jaydasht and Do-Ghalat (28°48′ N, 52°38ʹ E), are located in the central district of Firuzabad County, and the third village, Emam-shahr (28°27ʹ N, 53°07ʹ E), is located in the central district of Qir va Karzin County (Fig. 1). According to data obtained from the Iran Veterinary Organization at the time of the study, the livestock population in these three districts was 5711. A minimal sample size of 136 was estimated (95% confidence interval [CI], 5% margin of error), based on studies conducted in other endemic countries with a 10% infection rate. No particular skin manifestation related to leishmaniosis was observed in any of the livestock tested. Blood samples (8 ml) from jugular veins of 181 livestock, consisting of 114 goats, 16 cattle, 49 sheep and two donkeys, were collected in tubes containing K2EDTA and stored in a cold box before being transferred to the laboratory. Plasma and buffy coat were separated from the blood on the same days and stored at − 20 °C until use.

Meanwhile, blood samples were collected from 30 goats, 30 sheep and 30 cattle in a non-endemic *Leishmania* region, Shiraz, to define the direct agglutination test (DAT) cut-off titer and to determine the validity of the qPCR assay. qPCR with kinetoplast DNA (kDNA) primers was used to detect the Leishmania kDNA and quantify the parasite load. In addition, bidirectional sequencing was used to identify the parasite species after performing conventional PCR with internal transcribed spacer 1 (ITS1) primers. In this study, infected livestock was defined as those with positive results in qPCR and DAT.

### Direct agglutination test

All plasma samples were assessed for antibodies against *Leishmania* spp*.* by DAT, according to Harith et al. [18]. In summary, sera were serially diluted twofold in DAT diluent (0.1 M 2-mercaptoethanol in saline [0.9%]) to make dilutions of 100–102,400. Antigen was dispensed to each well (50 µl/well), and the plates were gently rotated and left overnight at room temperature. The DAT titer cut-off point was determined using samples from non-endemic regions. As a positive control, a confirmed human VL serum was applied.

### Quantitative real-time PCR and conventional PCR

Buffy coats digested in 500 µl of lysis buffer (0.5% Tween 20, 0.5% Nonidet P-40, 10 mM NaOH, 10 mM Tris pH 7.2) containing 320 mg proteinase K/ml were incubated at 56 °C overnight. DNA was then extracted using the simplified phenol–chloroform extraction method [19].

The qPCR assay was performed using the TaqMan Gene Expression Assay (Applied Biosystems, Thermo Fisher Scientific, Waltham, MA, USA) with forward and reverse primers directed against kDNA (CTTTTCTGGTCCTCCGGGTAGG and CCACCCGGCCCTATTTTACACCAA, respectively) and FAM-labeled TaqMan probe (TTTTCGCAGAACGCCCCTACCCGC-BHQ1) [20]. The Applied Biosystems 7500 Real-Time PCR System was used for amplification and detection, with the following reaction conditions applied: 2 min at 50 °C, 10 min at 95 °C, followed by a two-step temperature (94 °C and 60 °C) cycling for 45 cycles. The qPCR assays were performed with 5 µl of DNA in a final reaction volume of 25 µl. The standard curve was created using *Leishmania* DNA extracted from 5 × 10^6^ parasites, with serial dilutions of 5 µl, from 50,000 to 0.005 parasites prepared for each reaction tube. From among the samples that tested positive, we randomly chose 15 samples for conventional PCR using ITS1 primers to amplify the* ITS1* gene of *Leishmania* species. Conventional PCR was done according to Tai et al. [21]. Four PCR products were randomly chosen and sent for bidirectional sequencing using ITS1 primers to confirm the amplified DNA corresponding to *Leishmania* species.

### Statistical analysis

The Spearman correlation test was used to determine a correlation between DAT titer and parasite load, using SPSS version 18 software (SPSS IBM Corp., Armonk, NY, USA). The level of agreement between qPCR and DAT was determined by calculating Cohen’s Kappa (*k*) and interpreted as follows: negligible (*k* = 0–0.20), weak (*k* = 0.21–0.40), moderate (*k* = 0.41–0.60), good (*k* = 0.61–0.80) and excellent (*k* = 0.81–1).

## Results

A titer > 1600 was designated as the cut-off based on the results of the samples from non-endemic regions, which was also previously used for a study on livestock by Mukhtar et al. [22]. We detected *Leishmania* antibody at a titer > 1600 in 38.7% (70/181) (95% CI: 31.5–46.2) of the livestock tested, whereas *Leishmania* DNA was detected in 51.4% (93/181) (95% CI 43.9–58.9) of the livestock tested. DAT revealed that 11.5, 23.8 and 3.9% of the sheep, goats and cattle, respectively, were seropositive for infection with *Leishmania*. The titer distribution was: 36 animals with titer 3200, 25 with titer 6400, five with titer 12,800, one with titer 25,600 and three with titer 51,200.

According to the Spearman correlation test analysis, there was a significant relationship between the titer determined with the DAT and parasite load (*P* < 0.5). Three samples with high titers according to the DAT (51,200) were among those with the highest parasite load. Forty-two qPCR-positive samples which tested negative by the DAT had a low parasite load (1–40 parasites/ml; median: 7 parasites/ml). The level of agreement between the qPCR and DAT results was weak (*k* = 0.33; *P* < 0.000).

*Leishmania* DNA was found in 14.9, 29.3 and 6.6% of the sheep, goats and cattle, respectively, and in one of the two donkey’s buffy coat suspension. Among those samples with positive qPCR results, sample quantification yielded parasite loads ranging from 1 to 3000 parasites/ml (median: 100 parasite/ml). The 15 randomly selected samples with a positive qPCR result were also positive in the conventional PCR analyses using ITS1 primers.

Of the four ITS1-sequenced PCR products, two sequences revealed > 99% sequence identity with the ITS1 sequence of *L. infantum* with accession numbers MT497974 and AJ634370 (registered in the GenBank database). Of the remaining two products, one had an 86.32% sequence identity with the ITS1 sequence of *Leishmania* spp. with accession number MT302159, and the second had > 99% sequence identity with the ITS1 sequence of *L. major* with accession number AY260965 (both registered in the GenBank database). Of these four sequenced PCR products, two were related to cattle and two were related to sheep and goats. These four ITS1 DNA sequences were deposited in the GenBank database with accession numbers OM238068, OM238069, OM218660 and OM218661.

Both qPCR and DAT-positive results were found in 51 cases (28.2%). Tables 1 and 2 present the DAT and qPCR results in terms of livestock type, locations and tests.

**Table 1:** Direct agglutination test and qPCR results in different livestock from three districts of southern Iran

County	Village	Livestock (*n*)	DAT cut-off > 1:1600		qPCR	
			Positive,* n* (%)	Negative,* n* (%)	Positive* n* (%)	Negative* n*. (%)
Firuzabad	Jaydasht	Sheep (22)	9 (40.9)	13 (59.1)	15 (68.2)	7 (31.8)
		Cattle (10)	4 (40)	6 (60)	9 (90)	1 (10)
	Do-Ghalat	Sheep (18)	8 (44.4)	10 (55.6)	8 (44.4)	10 (55.6)
		Goat (40)	17 (42.5)	23 (57.5)	18 (45)	22 (55)
		Donkey (2)	0	2 (100)	1 (50)	1 (50)
Qir va Karzin	Emam-shahr	Sheep (9)	3 (33.3)	6 (66.7)	4 (44.4)	5 (55.6)
		Goat (74)	26 (35.1)	48 (64.9)	35 (47.3)	39 (52.7)
		Cattle (6)	3 (50)	3 (50)	3 (50)	3 (50)
Total		181	70 (38.7)	111 (61.3)	93 (51.4)	88 (48.6)

*DAT* Direct agglutination test

**Table 2:** qPCR and DAT results on the samples collected from livestock in southern Iran

Animal tested	*n* animals tested	DAT, *n* (%)	qPCR, *n* (%)	DAT+/qPCR+ results, *n* (%)	DAT+/qPCR− results, *n* (%)	DAT−/qPCR+ results, *n* (%)
Cattle	16	7 (43.75)	12 (75)	6 (37.5)	1 (6.25)	6 (37.5)
Sheep	49	20 (40.1)	27 (55)	20 (40.7)	0 (0)	7 (14.3)
Goat	114	43 (37.7)	53 (46.5)	25 (21.8)	18 (15.7)	28 (24.6)
Donkey	2	0 (0)	1 (50)	0 (0)	0 (0)	1 (50)
Total	181	70 (38.7)	93 (51.4)	51(28.2)	19 (10.5)	2 (23.2)

## Discussion

Firuzabad and Qir va Karzin counties in Fars province are endemic areas where both CL and VL are known to be actively transmitted. In Iran, dogs and rodents are the only confirmed primary reservoirs for VL and CL infections caused by *L. infantum* and *L. major*, respectively.

A few studies in Iran have found leishmaniosis in cats using both serological and molecular approaches, while the relevance of cats in the zoonotic transmission is still controversial [23]. In Iran’s VL and CL endemic regions, no attempts have been made to identify *Leishmania* infection in livestock that come into close contact with the principal reservoir host and graze near resting habitats of *Leishmania*-infected sand flies. In the present study, we found that 28.2% of the livestock tested were positive for *Leishmania*. It is unclear how these animals became infected, but given the importance of phlebotomine sand flies in the *Leishmania* infection cycle, it is likely that bites from infectious sand flies were the only route of transmission. Since dogs and rodents are the main reservoirs for VL and CL, and given the large population of rodents in these areas, as well as the presence of guard dogs for domestic herds, and assuming no preference for blood meal source for female sand flies, such livestock must be bitten by sand flies frequently and become easily infected.

 Yard et al. found that the majority of blood meals consumed by sand flies in the VL endemic region of northeastern Ethiopia were from donkeys (33.9%), followed by cows (24.2%), humans (17.6%), dogs (11.8%) and goats or sheep (8.6%) [17]. Abbate et al. reported that the most prevalent blood sources observed in sand flies in the *Leishmania* endemic area of Sicily came from wild rabbits (*n* = 28), humans (*n* = 24), goats (*n* = 16), horses (*n* = 13), pigs (*n* = 9), dogs (*n* = 9), chickens (*n* = 3), cows (*n* = 3), cats (*n* = 1), donkeys (*n* = 1) and rats (*n* = 1) [24]. A remarkably high bovine blood feed (92%) was reported by Gebre-Michael et al. in their analyses of blood meals of 273 fresh-fed *Phlebotomus orientalis* females from northwest Ethiopia [25].

It should be noted that in these three studies, the authors noted that a considerable percentage of phlebotomine blood meals came from livestock.

An animal is considered to be a reservoir of *Leishmania* infection when: (i) it is long-lived and is present in relative abundance; (ii) it is responsible for the long-term maintenance of parasites; (iii) it remains infected and apparently healthy for a long time; (iv) it bears high levels of parasites in the superficial skin vessels for availability to sand flies; and (v) sand flies depend on feeding on them [5, 6].

In VL, the parasites spread through skin macrophages and affect organs such as lymph nodes, bone marrow and spleen. Therefore, biopsies of these tissues for direct parasite detection are required to confirm parasite multiplication and survival in these organs. It is also recommended that samples be taken from the infected animals at different intervals to see how long they can stay infected. Another option is to look into the blood meal source of *Leishmania*-infected sand flies in these regions. Consequently, consideration of these mammals as reservoirs demands adequate and consistent evidence. Performing xenodiagnoses on cases with high parasite burdens is crucial to proving that these mammals are infectious to laboratory-reared sand flies.

To determine whether the same parasite clone is present, the *Leishmania ITS1* DNA sequences of VL and CL patients in this study area should be compared to that extracted from infected dogs, rodents, sand flies and livestock. Several previous studies in *Leishmania* endemic regions around the world have reported *Leishmania* infection in livestock, consistent with present study findings, although these studies differ in terms of sample size, diagnostic method applied, types of samples, presence of heterogeneous parasite populations and immune responses to the *Leishmania* parasite [12–14, 16, 26]. In the present study, some samples were positive by both molecular and serological methods (51/181; 28.2%), while others were positive by only one method. Our data show a direct correlation between parasite load and antibody titer determined by DAT; accordingly, some qPCR-positive samples with a low parasite load were negative by DAT (42/181; 23.2%), suggesting the onset of infection. According to a recent study, qPCR has a higher sensitivity than serological tests in identifying asymptomatic leishmaniasis in whole blood; however, the qPCR test could not detect all infected cases [27, 28]. Although it is unclear whether these results might be extended to the diagnosis of the infection with *Leishmania* in livestock, a combination of several methods may be the best strategy to diagnose *Leishmania* infection in livestock [28].

DAT results were positive in 38.7% (70/181) of the livestock tested, with approximately 10.5% (19/181) of the livestock being only positive by DAT, and the rest positive by both techniques. The antibodies detected in this 10.5% of the livestock may be associated with an old infection; nevertheless, the antigenic similarity between *Leishmania* and *Trypanosoma* infecting livestock should not be overlooked [29].

More than half (54%) of CL cases in WHO Eastern Mediterranean Region countries occur in Iran, with a quarter of these cases occurring in Fars province, with the predominant form of zoonotic CL caused by *L. major* [3, 30]*. *In the current investigation, the sequence of one of the randomly selected cases was *L. major*. The high percentage of infected livestock in this study is significant, emphasizing the need to pay attention to such animals for their ability to maintain and transmit *Leishmania* parasites.

Given the data above and the lack of clinical manifestations in the studied livestock and their large population, these animals may serve as a potential *Leishmania* reservoir. However, further research is needed to determine not only how these animals are infected and but also the feeding behavior of the sand fly in order to clarify the sand fly’s role in the epidemiology of *Leishmania* infection.

## Data Availability

Data supporting the conclusions of this article are included within the article. The raw data obtained during the present study are available upon request.

## Abbreviations

CL: Cutaneous leishmaniasis
DAT: Direct agglutination test
ITS1: Internal transcribed spacer 1
VL: Visceral leishmaniasis
